# Balancing Robustness against the Dangers of Multiple Attractors in a Hopfield-Type Model of Biological Attractors

**DOI:** 10.1371/journal.pone.0014413

**Published:** 2010-12-22

**Authors:** Ron C. Anafi, Jason H. T. Bates

**Affiliations:** 1 Division of Sleep Medicine, University of Pennsylvania, Philadelphia, Pennsylvania, United States of America; 2 Department of Medicine, University of Vermont, Burlington, Vermont, United States of America; Virginia Tech, United States of America

## Abstract

**Background:**

Many chronic human diseases are of unclear origin, and persist long beyond any known insult or instigating factor. These diseases may represent a structurally normal biologic network that has become trapped within the basin of an abnormal attractor.

**Methodology/Principal Findings:**

We used the Hopfield net as the archetypical example of a dynamic biological network. By progressively removing the links of fully connected Hopfield nets, we found that a designated attractor of the nets could still be supported until only slightly more than 1 link per node remained. As the number of links approached this minimum value, the rate of convergence to this attractor from an arbitrary starting state increased dramatically. Furthermore, with more than about twice the minimum of links, the net became increasingly able to support a second attractor.

**Conclusions/Significance:**

We speculate that homeostatic biological networks may have evolved to assume a degree of connectivity that balances robustness and agility against the dangers of becoming trapped in an abnormal attractor.

## Introduction

Biological control networks share many formal similarities with artificial neural networks [1], [2], [3], [4], [5]. In particular, the Hopfield net is a recurrent type of neural network with a dynamic state defined at any instant by the set of output levels at each of its nodes This state moves around on a multi-dimensional energy landscape having one or more local minima that act as attractors for states located nearby. Through appropriate adjustment of the weights of the links between the nodes (the analog of synaptic strengths between real neurons), the Hopfield net can differentiate between classes of initial state based on the particular attractors they converge toward [6]. This makes the Hopfield net suited for performing associative or content addressable memory tasks. Real neuronal networks actually appear to have more limited connectivity, but small world Hopfield nets can also have multiple attractors [7], [8], [9], [10]. The nonlinear summing junctions and variable link weights of the Hopfield net thus embody what many consider to be the essential information-processing elements of networks of real biological neurons. We are concerned here, however, with the relevance of the Hopfield net for non-neuronal biological networks, such as those pertaining to metabolism or gene transcription, and which have also been shown to have the ubiquitous small world topology [11], [12].

Important for the modeling of general biological networks is the fact that the functional attributes of the Hopfield net are not contingent upon the nonlinear characteristics of the nodes being step functions [13]. In fact, any suitably saturating nonlinearity will do. In particular that small-world networks based on the Hopfield architecture can have multiple attractors when their nodal nonlinearities conform to the Michaelis-Menten equation frequently encountered in biochemical reaction kinetics [3]. Like biological networks, Hopfield nets contain numerous excitatory links. Thus, any real world implementation of these networks must consume energy, which is an essential requirement for all biological systems in order that they maintain a state far from thermodynamic equilibrium [14], [15]. Hopfield nets thus share some key operational characteristics in common with biological systems. Furthermore, in contrast to simple analogue control systems that create directed restoring forces designed to return a system to a pre-programmed set point, Hopfield nets exhibit attractor dynamics while at the same time reflecting the complexity of biological systems.

Here, however, we encounter an intriguing dichotomy. When using a Hopfield net in its classic application related to content addressable memory tasks, a key design goal is to maximize the number of distinct attractors in the net's energy landscape, while keeping their basins of attraction as deep as possible. This combination allows optimal discrimination among distinct attractors and thereby maximizes the number of distinct entities that the net can “remember” reliably [6]. By contrast, one of the fundamental requirements for living systems is to be able to maintain homeostasis in the face of varied and ongoing environmental inputs, often of a noxious variety. Continued health depends on the system's ability to mount an appropriate response to such inputs and, subsequently, to return toward a state of normality regardless of what regions of the energy landscape its state had to visit in the meantime. A functional biochemical interaction network would thus seem to be best served by an energy landscape consisting of a single large basin of attraction that funnels all aberrant states toward a single attractor corresponding to the “normal state” of the network. The alternative (i.e. having more than one attractor) would seem to pose the risk of having a biochemically normal network become functionally entrapped in a “pathological attractor”, should it receive the right stimulus.

We thus face two possibilities for biological networks. One is that multiple attractors do exist for such networks, in which case we have to deal with the possibility of entrapment in a non-normal attractor [3], [5]. It is not clear whether or not this actually happens in living systems, but if it does it might explain the existence of some of the many chronic diseases currently labeled as “idiopathic” [3] in which the body seems to operate in an abnormal fashion for no obvious reason. In fact, the theory of networks as applied to the immune system is already well developed [1], and a multiple-attractor Hopfield-type theory has been invoked to explain the effects of vaccination on immunological status [5]. In a similar vein, Kauffman has long posited that the various cellular phenotypes found in the body correspond to attractors in the landscape defined by the network of gene interactions [4], [16] and that, given sufficiently large perturbations, cells can be moved so dramatically from their normal attractor that cell type itself can be changed [17].

The other possibility for networks in living systems is that they are dominated by single attractors, in which case we have to ask how this could be. Given that multiple attractors can potentially exist in large networks governed by the Michaelis-Menten nonlinearity [3], the question arises as to what structural constraints must be in place to ensure only one attractor. It is this question that we attempt to probe herein. We present evidence that there is a critical degree of network connectivity beyond which multiple attractors can be supported, and suggest that the connectivity of real biological networks may have evolved to balance the dangers of multiple attractors against the need for agility and robustness.

## Results

We first investigated how the existence of a single attractor in a Hopfield-type network is influenced by its connectivity. Following the methods of Hopfield, [1] we began with several fully connected networks with *N* equal to 50, 100, 200, or 400 nodes. Each network was constructed so as to have only a single designated attractor. We then randomly eliminated inter-nodal connections, each time checking to see if the designated attractor remained intact. We checked for convergence to the designated attractor from 100 different, randomly chosen initial states. The net was iterated 500 times from each initial state or until it converged to either a steady state or a limit cycle up to period 4. (In eliminating individual links, the symmetry of the initially fully connected net was lost along with the Hopfield structure that guarantees the existence of only fixed-point attractors, thereby allowing for the possibility of limit cycles). If the net converged toward the designated attractor for all initial conditions, the links were permanently eliminated. Otherwise they were reinstated and the elimination of a different set of links was assessed.

**Figure 1:**
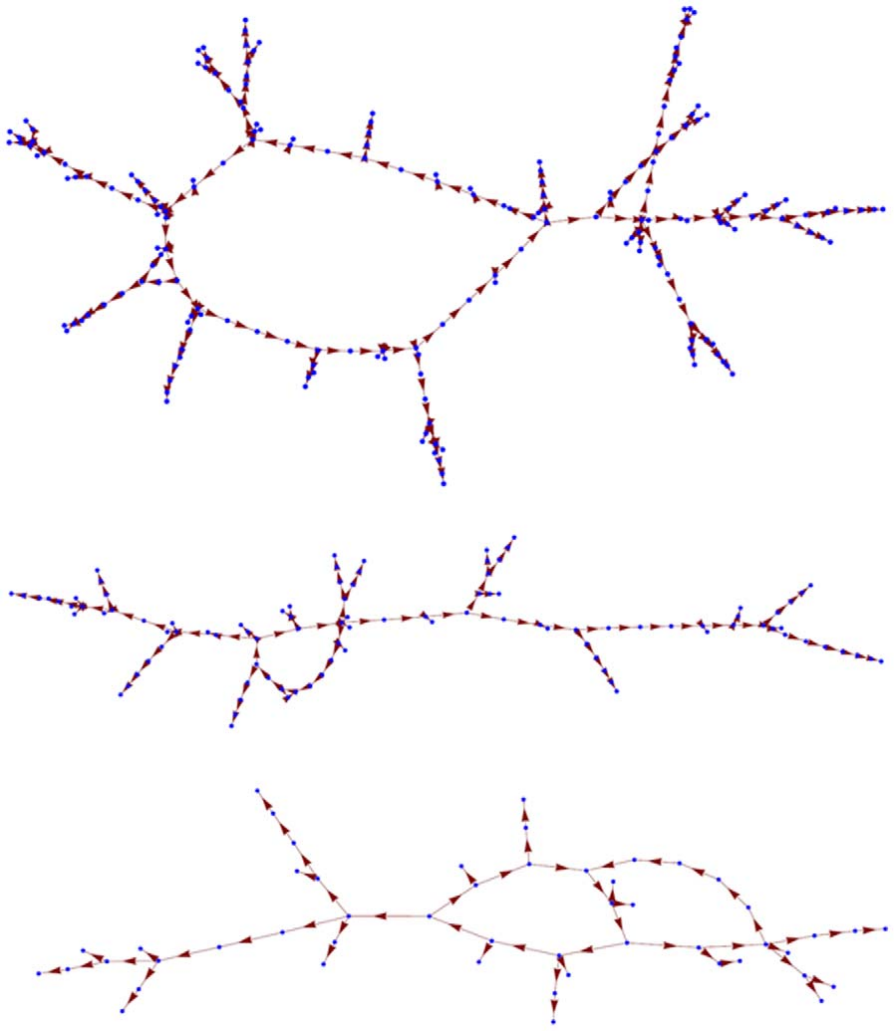
The final configurations (nodes and their directed interconnections) of randomly generated Hopfield nets having 200 (top), 100 (middle) and 50 (bottom) nodes. These nets began fully connected with their link weights chosen to define a single attractor (the designated attractor). Links were removed until the nets were no longer able to support their designated attractors. The configurations shown are those with the fewest connections that can support each attractor.

**Figure 2:**
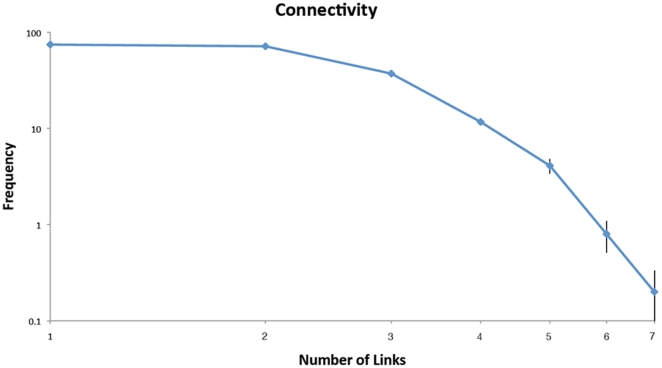
Histogram showing frequency (the number of nodes having a given number of links both arriving and leaving) versus number of links for a fully pruned 200-node net (average of 10 independent runs).

Figure 1 shows examples of fully pruned networks that retained the ability to converge to their respective designated attractors. These final configurations show some degree of variation, but in terms of overall structure they share strong similarities. In particular, the network configurations shown in Fig. 1 are very sparse with few circular pathways. Indeed, while a network with *N* nodes starts with *N* ^2^ connection, we were able to reduce this number to only slightly more than *N* while retaining the designated attractor. Most of the nodes thus end up receiving very few connections, ranging from 1 to 7. To characterize this connectivity more fully, we pruned 10 different 200-node networks and found that the final network configurations had between 201 and 207 connections in total. Furthermore, the frequency distribution of nodes as a function of the number of connections decreased monotonically (Fig. 2). This is reminiscent, both in shape and magnitude, of the small world connectivity distributions that have been widely reported for metabolic and genetic networks [12], [18], [19].

**Figure 3:**
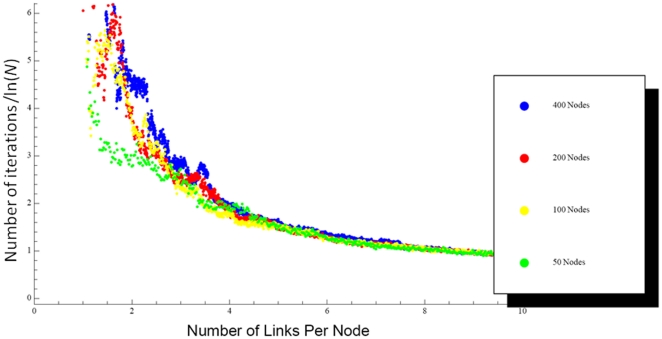
Number of iterations of the Hopfield net required to achieve convergence to the designated attractor from an arbitrary starting state as a function of the number of links per node in the net, for nets of *N* = 50, 100, 200 and 400 nodes. The vertical axis has been normalized by ln(*N*).

By design, the final network configurations shown in Fig. 1 are extremely fragile in the sense that elimination of even one more link will destroy the designated attractor. In contrast, random elimination of 95% of the original full set of links usually left the designated attractor intact. This illustrates a powerful tradeoff between parsimony and robustness. The cost of parsimony is more than just fragility, however, as there is also a tradeoff against the speed with which the network can respond to external perturbations [7]. As the links were pruned from the network, the average number of iterations they required to converge to the designated attractor from a random starting state increased, rising dramatically as the number of links neared the minimum value; Fig. 3 shows that the number of iterations increases sharply as the average number of links per node drops below about 3. The rate of convergence was also found to scale with the natural logarithm of the network size (*N*). Thus, when the number of iterations required for convergence is normalized by ln(*N*) and plotted against the mean number of links per node, a universal curve emerges (Fig. 3). With this empirical scaling law we can extrapolate our result to networks of arbitrary size.

We also evaluated how changes in network connectivity impacted the ability of the network to support a second attractor, and identified another tradeoff that may have important implications for chronic disease. This relates to the ability of the network to support a second attractor. Although we set up our networks so that each had only a single designated attractor from the outset, a fully connected Hopfield net can support multiple attractors if its link weights are chosen appropriately [6], [13]. Indeed, the maximum storage capacity of a fully connected *N*-node Hopfield net is in the order of *N*/ln(*N*) [6]. On the other hand, the fragile networks shown in Fig. 1 are unable to sustain any more than their single designated attractors. Therefore, somewhere between the fully connected condition and the sparse connectivities illustrated in Fig. 1 is the point where these nets are able to support a second attractor.

**Figure 4:**
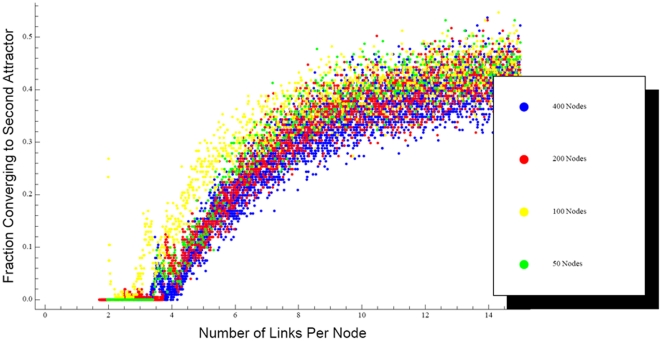
Fraction of times that networks converged to a second orthogonal attractor versus the number of links per node in the network, for nets of *N* = 50, 100, 200 and 400 nodes. The minimal set of links previously determined to support the first designated attractor in each net were not affected during the link pruning process.

We searched for the second attractor point by adding several thousand links back to individual examples of fully pruned networks (such as those shown in Fig. 1), with the weights of these new links adjusted so that the network supported both the original designated attractor and a second designated attractor that was orthogonal to the first. We then randomly removed these new links without touching the minimal set of links previously found necessary to support the first attractor. At each step in this process, the network was launched from 200 random initial configurations, all of which were required to converge to one or other of the two designated attractors before the links were permanently deleted. As the new links were pruned, the fraction of the runs that converged to the second attractor decreased (Fig. 4). These results also scaled with network size so that, regardless of *N*, the chance of convergence to the second attractor became extremely small when the network was reduced to approximately 3*N* connections. When fewer than about 2 connections per node remained, the second designated attractor was no longer supported as a fixed point of the system. There thus appears to be a range of networks connectivities between 1 to 2 connections per node that can support a single designated attractor but not a second one. This degree of sparsity, however, comes at the cost of both network fragility and speed, as Fig. 3 indicates a sharp decline in convergence speed as connectivity falls below 3 connections per node.

## Discussion

Biological networks are sparse, each agent communicating directly with only a very few of its possible counterparts [12], [18], [19]. Our minimal networks (Fig. 1) are also sparse, and have connectivity distributions (e.g. Fig. 2) that decrease monotonically, reminiscent of a number of biological networks [12]. Our results thus suggest that a sparse small-world network is suited to operating within a dynamic basic of attraction. This would allow the network to always return toward normality following the continual and varied perturbations it receives from the environment. These networks thus accomplish homeostatic control in a manner that resembles true biological systems more closely than do *ad hoc* control models based on a pre-programmed restoring force. One the other hand, the sparseness of these networks obviously mitigates against robustness to damage. The minimal networks shown in Fig. 1 are fragile to the extent that elimination of a single link will render each of them unable to support their designated attractor. This would seem to be a precarious position to be in from the point of view of survival. Biologic networks would do well to have at least some redundancy built into their structures by having more than the minimal set of links required to support the dynamic attractor corresponding to health; one can imagine that minimally connected networks would not have fared well in the contest for natural selection. Indeed, the actual degree of sparseness seen in naturally occurring networks has been suggested to result from evolutionary forces construing to produce a balance between robustness and parsimony [20].

We have identified in the present study, however, two other aspects of sparseness that have opposing advantages, and which we speculate have to be balanced in some way by biological networks. One of the tradeoffs we found is illustrated in Fig. 3, which shows that a higher density of links enables a network to return more rapidly toward its attractor state following a perturbation. This obviously bodes well for the ongoing maintenance of homeostasis, and so might be considered another factor giving survival advantage to an increase in the link density of a biologic network. On the other hand, as the link density increases above the minimum level required to maintain a given attractor state, there comes a point at which it is able to support a second attractor (Fig. 4). The requirement that a biological network be both robust and agile may thus require a degree of connectivity that supports the existence of aberrant or pathologic attractors. In such a situation, an inopportune interaction with the environment could potentially drive the network into the clutches of an abnormal attractor.

Of course, the conclusions drawn in this study are based on an idealized model that is highly simplified relative to real biological systems. We have assumed, for example, that homeostasis is characterized by a single “normal” attractor, and that all other attractors are pathologic in some way. It is likely that normality in a real biological network is defined by a cluster of attractors that allow for flexibility in dealing with different environmental conditions or developmental stages. If such attractors are localized in some sense, then a pathologic attractor could likely be defined by its distance away from the normal cluster. Furthermore, in probing for the existence of multiple network attractors, we experimented with random environmental perturbations (i.e. initial states) drawn from a uniform probability distribution. In reality, the perturbations impinging on an organism are more likely to follow a probability distribution having some central tendency determined by the particular environment that the organism lives in. Such a biological network could exist comfortably with an aberrant attractor if the vast majority of perturbations it is likely to experience remain within the neighborhood of the normal attractor. Finally, the behavior of the nodes in our networks was characterized by step-functions, as in the classic Hopfield. Such nets are straightforward to set up, and may even be amenable to analytical investigation [21]. Of course, actual metabolic or genetic networks almost certainly involve more complicated nodal decision functions, the Michaelis-Menten equation perhaps being the simplest possibility [3], so our networks are not linked to any particular biological system but rather are designed to embody the essential features which we believe ought to be found in any biological network. These important details might allow the “sweet spot” for network connectivity to be somewhat larger than Fig. 4 implies. These caveats, however, do not change the overall message of our study, which is that the density of biological network links may be an important evolutionary design feature that balances speed and flexibility against stability.

We thus speculate that functional entrapment of a structurally normal biological network in an abnormal attractor might underlie the pathogenesis of some chronic disease states. We do not yet know which, if any, real diseases result from this type of process. However, if our speculation is true then it has certain important and sobering implications. First, while there would be clear functional evidence of disease when a biologic network succumbs to an abnormal attractor, the structure of the network and its individual components would remain completely normal. Furthermore, the instigating factor that pushed the network out of its normal basin of attraction might be long gone by the time the disease is investigated, contributing to the mystery surrounding its nature and origins. Trying to treat a disease of this nature might also be problematic, as it would require applying just the right combination of perturbations to push the network back into its normal attractor. One approach to achieving this might be to apply large random perturbations that, in analogy with the numerical technique known as simulated annealing [22], could cause the system to eventually find its way back to normality by chance. In any case, there remains much to be done to establish the validity of this theory, which will likely require an improved understanding of the signaling and/or metabolic pathways involved in candidate chronic diseases. One such disease might be idiopathic pulmonary fibrosis, which manifests as the inappropriate progression of processes in the lung that are normally only seen transiently in the repair of tissue injury [23]. Chronic insomnia also shares features with this disease model. Insomnia is often precipitated by an acute event but may persist for years after the resolution of the inciting stressor [24]. Indeed one of the most effective treatments, sleep restriction therapy, is a controlled form of partial sleep deprivation [25] and may be viewed as an attempt to provide a sufficiently large perturbation in sleep-wake dynamics so as to move the patient back into the basin of attraction corresponding to the normal state. Certain types of autoimmune disease and cancer might also be usefully viewed from the perspective of entrapment in an aberrant attractor. In any case, we believe this may be a direction worth pursuing, in view of the number of chronic diseases that remain recalcitrant to understanding and treatment.

## Materials and Methods

The model development and computations described below were performed using Mathematica™ (Wolfram Research Inc., Champaign, IL).

The Hopfield net is a recurrent type of artificial neural network consisting of an array of nodes, each of which links directly to every other node, and possibly also to itself [6], [13]. The nodes are nonlinear summing junctions that receive input from all other nodes, with each input being weighted by a strength associated with the corresponding incoming link. The links are symmetric in the sense that the link strength from node *a* to node *b* is the same from *b* to *a*. Also, these links may be positive (excitatory) or negative (inhibitory). Each node has a value that marks it as is either quiescent (value  = −1) or active (value  = 1) based on whether the sum of the weighted inputs the node receives from the other nodes exceeds a certain threshold (taken to be zero in the present case). The network is recursively iterated by multiplying the value of each node by the strengths of its links to the other nodes in order to create inputs to these other nodes at the next time step. The state of a Hopfield net, defined by the set of values of each of its nodes, defines an energy landscape having one or more local minima. As the net is iterated, its state will converge from a given starting point toward a stable attractor that sits at the bottom of the basin of attraction in which the initial state is located [6], [13]. In general, the energy landscape of a Hopfield net contains numerous attractors that each act as collection points for states located nearby. Importantly, the locations of the attractors in the energy landscape are determined by the link strengths between the nodes, and these strengths can be chosen to place the attractors at desired locations. The Hopfield net can thus be used to differentiate between classes of initial state on the basis of the particular attractors the various states converge toward. This allows the Hopfield net to function as an content addressable memory device in which the different attractors may correspond to particular objects of interest [6].

We began with a fully connected Hopfield networks with *N* equal to 50, 100, 200 or 400 nodes. Each node had a value of either −1 or 1 depending on whether its inputs summed to <0 or ≥0, respectively. The weights of the links were assigned according to the algorithm described by Hopfield [13] so that there was only a single attractor state, which we call the designated attractor. The designated attractor of a network was specified as follows. A *N*-dimensional vector **s** was generated by randomly assigning to each of its component, *s_i_*, a value of −1 or 1. The link strengths of the connectivity matrix **T** were then chosen to make this vector the single designated attractor of the network, following Hopfield [13], by taking **T** to be the dyad product of **s** with itself. That is,
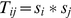
(1)


This causes **T** to have a single non-zero eigenvalue associated with the eigenvector **s**. Furthermore, for any vector **x**, the inner product **T⋅x** is either the null vector or a vector in the direction of **s**. (Note that the complement of **T** is also an attractor of the network, but we will focus here only on **T** itself, as there is no guarantee that its complement in a real biological system would be physically or physiologically reachable.)

The networks were iterated as follows. Starting from a random initial state vector **x[**0**]**, the state of the system at time step *n*, denoted by **x[** *n* **]**, was generated, again following Hopfield [13], as
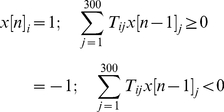
(2)


The system was iterated 500 times or until it converged to a fixed point. For networks that had been pruned and thereby lost their initial symmetry, we also evaluated for convergence to a stable limit cycle of period 4 or below. This process was repeated for each of 200 randomly generated **x[**0**]**, and the existence of the designated attractor was taken as confirmed if the net converged to this attractor for all initial conditions. We also recorded the average number of iterations required for the system to converge to the designated attractor. We then removed the vast majority of connection so that on average there remained only 10 connections per node. Connections were removed by setting the corresponding *T_ij_* values to 0, and networks were re-tested for the persistence of the designated attractor in the reduced configuration. Invariably, this sharp reduction in network size did not impact the continued existence of the single attractor state. We then randomly removed one connection at a time. At each step in this removal process the network was probed, starting from 200 random initial states, for the continued existence and global stability of its designated attractor. If one or more of the initial states failed to converge to the designated attractor at any step, the connections that had been removed at the previous step were reinstated and a different set of random connections was removed. The configuration of the network during the pruning process was saved periodically for later evaluation. The connectivity matrix describing the minimal set of connections necessary to preserve the functioning of the network was denoted T^*^. Figure 1 demonstrates a plot of representative examples of the T* networks that emerged from the process using different sized starting networks. This process was then repeated for 10, 200 node networks each with a distinct randomly chosen designated attractor. Figure 2 demonstrates the a histogram of network connectivity (shown as mean +/− Standard Error).

In order to test the ability of the network to support a second attractor, a state vector **r** was randomly generated subject to the constraint that it be orthogonal to **s,** such that **r⋅s = 0**. Connections were added to T^*^ so that **s** and **r** became attractors for the network with a combined basin of attraction spanning the whole domain. The new connectivity matrix U was constructed through a modification of Hopfield's original formulation as follows: 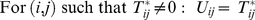


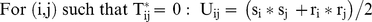



Thus the connectivity matrix **U** differed from the original Hopfield formulation for a network with attractors r and s, only at the approximately *N* of the *N* squared connections inherited from T^*^.

The fully connected network was tested with 1000 randomly generated initial conditions to ensure that the network would eventually converge to one of the two designated attractors. The connections that had been added to the skeleton of T^*^ were then randomly pruned. We initially removed the vast majority of connection so that, on average, only 15 connections per node remained by setting the remaining *U_ij_* values to 0. Individual connections were then randomly selected one connection at a time for sequential deletion. At each step, we tested to ensure that both **s** and **r** remained fixed points of the network, and we by launched it from 200 randomly generated initial configurations to ensure that their combined basins of attraction spanned the configuration space. All starting configurations were required to converge to one of the designated attractors before the links were permanently deleted. Both the average number of iterations required for the network to converge and the fraction of initial conditions that converged to each of the two designated attractors was tabulated for each configuration.

Mathematica™ codes for performing the above calculations are included in the supporting information (Appendixes S2 and S3). The codes are explained in Appendix S1.

## Supporting Information

Appendix S1 (0.02 MB DOCX)

Appendix S2 Network Pruning Mathematica code
(0.11 MB DOCX)

Appendix S3 Dual attractor network Mathematica code
(0.09 MB PDF)
