# Supplementary material for: Balancing Robustness against the Dangers of Multiple Attractors in a Hopfield-Type Model of Biological Attractors
Source: PLoS One. 2010 Dec 22;5(12):e14413. doi: 10.1371/journal.pone.0014413 (PMC3008716; doi:10.1371/journal.pone.0014413)
Supplement: Appendix S1 — (0.02 MB DOCX) [file pone.0014413.s001.docx]

**Online Supplemental Materials**

Here we provide the Mathematica code used in these simulations and the output produced. *SparseNetwork* illustrates the methods used to generate the Hopfield mapping function and prune the connectivity matrix. *DualAttractorNetwork* takes a skeleton network generated as the output from *SparseNetwork* and adds the required connections for the network to have a 2^nd^ attractor before again pruning the network.
