# Supplementary material for: Balancing Robustness against the Dangers of Multiple Attractors in a Hopfield-Type Model of Biological Attractors
Source: PLoS One. 2010 Dec 22;5(12):e14413. doi: 10.1371/journal.pone.0014413 (PMC3008716; doi:10.1371/journal.pone.0014413)
Supplement: Appendix S2 — Network Pruning Mathematica code (0.11 MB DOCX) [file pone.0014413.s002.pdf]

Preliminaries

In[1]:=
```
ClearAll[t, x, y, g, Size, Sr, Si, outx, outy, Size]
(ldir = "/Users/ron/Hoppfield/Rev2a";
 SetDirectory[ldir])
```

Out[2]= /Users/ron/Hoppfield/Rev2a

In[3]:=
```
speed = {};
Size = 25;                (*specify the size of the network - number of nodes*)
Sz = ToString[Size]
(*create a string version of this size specification for file names*)
SetAttributes[g, Listable]
(*allows the function g to be applied to elements of a vector*)
g[x_] := 1 /; x >= 0
(* the function g is a defined to be a specialized step function*)
g[x_] := -1 /; x < 0

Hop[T_, x_] := g[T.x]       (*The defines a single application of the hopfield map *)

(*with a network matrix denoted  T applied to a input vector x*)

(*with a step function then applied to the output vector*)

Error[x_, y_] := Min[(x - y).(x - y), (x + y).(x + y)] (*This finds the number of differences*)
                                                        (*between two state vectors x and y*)

(*recognizing the x and -x are the same*)

Correct[x_, y_] := 0 /; Error[x, y] == 0;
(*the vectors x and y are the same if error=0*)
Correct[x_, y_] := 1 /; Error[x, y] != 0;

Sr = Table[RandomReal[{-1, 1}], {i, 1, Size}];
Si = g[Sr];
(*generate a random vector Si composed of +1'-1's*)
                                     (*designated to be the attractor of the network*)

t = Outer[Times, Si, Si];      (*compute a full connected connectivity matrix T *)

(*which is the dyad (outer) product of Si with itself*)

x[0] = Table[RandomChoice[{1, -1}], {i, 1, Size}];
(*demonstrate the convergence of the network  *)

(*applying it to a random initial vector x[0] *)
x[1] = g[x[0]];
Do[x[n] = Hop[t, x[n - 1]], {n, 2, 10}]
outx = Table[x[n], {n, 1, 5}];
MatrixPlot[outx, AspectRatio → .8, MaxPlotPoints → Size^2](* visualize the results*)

(* each colum being blue or orange for +/-1*)

(* and each row being another application *)
                                                        (* of the hopfield mapping *)
```

First Remove Fixed Number of Links

In[16]:=

```
ConectChoices = Table[n, {n, 1, Size^2}];  (*create a list of removable connections*)
NumConections = Size^2;
tempt = t;
testgood = 1;                 (* a boolean variable will be set to 0 if the  *)
                              (* pruned network passes the test *)

avgspeed = 0;
testnum = 200;                (*number of random initial conditions to test*)
ittlimit = 500;               (*number of itterations to allow for convergence*)


numtokeep = Round[Size * 10];
(* number of connections to keep = average of 10 connection per node *)



While[testgood ≠ 0,
 (* continue looping untill a reduced network is found that*)
 {                               (* has a single
   attractor with global basin of attractor *)
  avgspeed = 0.,
  tempt = IdentityMatrix[Size] - IdentityMatrix[Size],
  (*Create an array of 0's of proper size*)

  (*that will later be used as a trial connection matrix*)

  TempChoices = RandomSample[ConectChoices, numtokeep],
  (*Randomly choose 5% of links to keep *)
  For[i = 0, i <= numtokeep - 1, i++
    (*Map each labeled choice to the *)
    {Choice = TempChoices[[i]],
     (*Coressponding i,j connection in T*)
     r1 = Quotient[Choice - 1, Size] + 1,
     r2 = Mod[Choice - 1, Size] + 1,
     tempt[[r1, r2]] = t[[r1, r2]]}
   (*For just these i,j maintain the nonzero links in T *)
  ],
  test = 1,
  etot = 0,
  While[(test ≤ testnum) && (etot == 0),
   (*Continue looping unless there has been an error or*)

   (*the required number of tests have been passed *)
   {test = test + 1,
    tempvector[0] = Table[RandomReal[{-1, 1}], {i, 1, Size}],
    (*Create random initial vector*)
```

```
        d = 1,                              (*d will be the distance between two subsequent *)
        n = 0,
        (*applications of the net, d=0 implies convergence *)
        While[(n < ittlimit) && (d > 0),    (*loop until the max
          number of iterations completed*)
                                            (*or until the sequence has converged *)
             {n = n + 1,
              tempvector[n] = Hop[tempt, tempvector[n - 1]],
              d = Error[tempvector[n], tempvector[n - 1]]
              }],
        etot = etot + Correct[tempvector[n - 1], Si],
        avgspeed = avgspeed + n - 1
        (*avg speed is the number of itterations +1, required for*)
       }],                              (*convergence. It is a
       running total and needs to be divided *)
                                        (*by the numbe of tests,
      to get the average num of itterations*)

      If[etot ≤ 0,
       (*if the network converged to the desingnated attractor for*)
       {t = tempt,                      (*all initial condition, accept change*)
        ConectChoices = TempChoices,  (*update list of links that may be deleted in future*)
        NumConections = Length[ConectChoices],
        testgood = 0},
       {testgood = 1, Print["had to try a different set"]}],
      (*if there were errors, the test failed and try a new set of deletions*)
      Print[etot, " ", NumConections]
     }]
    t = SparseArray[t];
    speed = {};
    speed = Join[speed, {{NumConections, avgspeed / testnum}}]
```

```
0 250
```

Out[27]= {{250, 2.68}}

Remove Random Links One-At-A Time: checking to see if link already tested and discounting links that are a node's only input

In[28]:=

```
RemovableChoices = ConectChoices;
(*A list of all connections that might still, at some point, be removable*)
TempChoices = RemovableChoices;
(*A list of connections that might be removable in the present network configuration*)

NumConections = Length[ConectChoices]; (*Number of total connections*)
NumTempConections = NumConections;
NumRemovableConections = NumConections;

ittlimit = 600;                        (*number of random initial conditions to test*)
testnum = 200;                         (*number of itterations to allow for convergence*)

While[NumTempConections > 0,     (*Continue looping until
  there are no more removable links in the present configuration*)
 {
  Choice = RandomChoice[TempChoices],
  (*Choose a random link from the list of TempChoices *)
  r1 = Quotient[Choice - 1, Size] + 1,      (*Find the i,
  j in the matrix that coorespond to this choice*)
  r2 = Mod[Choice - 1, Size] + 1,
  originalmatrixlement = t[[r1, r2]],
  (*Save the current value of that connectio (+/-1) in case it needs to be re-instated*)
  t[[r1, r2]] = 0,                          (*Set the link=o, ie break that connection*)
  etot = 0,
  If[(t[[r1]].t[[r1]] == 0) ,               (*if removing this link means that
    a node will no longer be connected to the network *)
   {etot = 100,                             (*we dont have to test,
    as we know it will fail*)
    RemovableChoices = Complement[RemovableChoices, {Choice}],
    (*remove this link from the list of connections that might ever be severed*)
    NumRemovableConections = NumRemovableConections - 1
   }],
  test = 1,                                  (*test counts the number
   of initial conditions that have been tried with the present config*)
  avgspeed = 0.,                            (*this will be used to
   keep track off the itterations required for convergence*)

  While[(test ≤ testnum) && (etot == 0),   (*continue to loop until
    the required number of tests have been run, or a test was failed*)
   {test = test + 1,
    tempvector[0] = Table[RandomReal[{-1, 1}], {i, 1, Size}],
    (*Choose a random initial condition/perturbation*)

    d = 3,
```

```
    (*d will be the distance between two consecutive itterations of the hopfield net *)
    n = 0,        (*d=0 would mean the output has converged to a steady state*)

    While[(n < ittlimit) && (d > 0),     (*Continue to itterate
      map until it has converged or reached max number of itterations*)
        {n = n + 1,
         tempvector[n] = Hop[t, tempvector[n - 1]],
         d = Error[tempvector[n], tempvector[n - 1]]
         }],
    avgspeed = avgspeed + n - 1,
    (*update the itterations so far required at this network confiugration*)
    etot = etot + Correct[tempvector[n - 1], Si], (*update the total number of errors*)
    If[d > 0, etot = 100]
    (*if the network didn't converge this is considered an error*)
   }],

  If[etot ≤ 0,                                  (*if the reduced net
    converged to the designated attractor (Si) for all initial conditions: *)
   {t[[r1, r2]] = 0,                            (*permanetly sever connection*)
    ConectChoices = Complement[ConectChoices, {Choice}],
    (*remove this link for the list of connections*)
    RemovableChoices = Complement[RemovableChoices, {Choice}],
    (*remove this link from the list of connections that might ever be severed*)
    TempChoices = RemovableChoices,
    (*all links in that list are now possible targets for removaal *)
    NumConections = NumConections - 1,
    (*reduce the number of connection, and removable connections by 1*)
    NumRemovableConections = NumRemovableConections - 1,
    NumTempConections = NumRemovableConections,
    speed = Join[speed, {{NumConections, avgspeed / testnum}}],
    (*appemd the # of links, and avg num of itterations required for convergence*)
    If[Mod[NumConections, 20] == 0, Print[NumConections]]
    (*after 20 links are deleted update the user*)
   },
   {                                                       (*if the
     reduced network did not converge to Si for all initial conditions: *)
    t[[r1, r2]] = originalmatrixlement,               (*restore the original link*)
    TempChoices = Complement[TempChoices, {Choice}],
    (*remove this link from the list of remval targets in the current network configu*)
    NumTempConections = NumTempConections - 1
   }
  ]

 }]
MatFilename = "Tmatrix" <> Sz <> ".mx"                (*Create file names
 to store the fully pruned Matrix,Convergence Speed, and Attractor*)
SpeedFilename = "TSpeed" <> Sz <> ".mx"
AttractorFilename = "Attractor" <> Sz <> ".mx"

DumpSave[MatFilename, t];                          (*Store data listed above for review*)
```

```
DumpSave[SpeedFilename, speed];
DumpSave[AttractorFilename, Si];
```

In[49]:=

```
ListPlot[N[speed], PlotRange → All, AxesOrigin → {0, 1}]
GraphPlot[Transpose[t], SelfLoopStyle → True, MultiedgeStyle → True,
 DirectedEdges → True, AspectRatio → 1, VertexLabeling → False]

x[0] = Table[RandomChoice[{1, -1}], {i, 1, Size}];
(*demonstrate the convergence of the network  *)

(*applying it to a random initial vector x[0] *)
x[1] = g[x[0]];
Do[x[n] = Hop[t, x[n - 1]], {n, 2, 120}]
outx = Table[x[n], {n, 1, 120}];
MatrixPlot[outx, AspectRatio → .8, MaxPlotPoints → Size^2](* visualize the results*)

(* each colum being blue or orange for +/-1*)

(* and each row being another application *)
                                                    (* of the hopfield mapping *)
```
