# Supplementary material for: Balancing Robustness against the Dangers of Multiple Attractors in a Hopfield-Type Model of Biological Attractors
Source: PLoS One. 2010 Dec 22;5(12):e14413. doi: 10.1371/journal.pone.0014413 (PMC3008716; doi:10.1371/journal.pone.0014413)
Supplement: Appendix S3 — Dual attractor network Mathematica code (0.09 MB PDF) [file pone.0014413.s003.pdf]

Get Old Sparse Connectivity Matrix (Results from Previous Simulation)

```
ClearAll[t, x, y, g, Size, Sr, Si, outx, outy, Size]
(ldir = "/Users/ron/Hoppfield/Rev2a";
 SetDirectory[ldir])

Size = 200;
Sz = ToString[Size]

Tfile = "Tmatrix" <> Sz <> ".mx"
AttractorFile = "Attractor" <> Sz <> ".mx"
Get[Tfile]
(*loads fully pruned connectivity matrix from previous simmulation*)
oldmatrix = t;          (*and stores as oldmatrix*)
Get[AttractorFile]      (*loads designated attractor Si*)

Size = Length[t]; (*number of nodes*)
```

Construct Needed Hopfield functions

```
twostatespeed = {};    (*initialize list to record speed of convergence*)

SetAttributes[g, Listable]
(*allows the function g to be applied to sepereate components of a vector*)
g[x_] := 1 /; x >= 0
(* the function g is a defined to be a specialized step function*)
g[x_] := -1 /; x < 0

Hop[T_, x_] := g[T.x]       (*The defines a single application of the hopfield map *)

(*with a network matrix denoted  T applied to a input vector x*)

(*with a step function then applied to the output vector*)

Error[x_, y_] := Min[(x - y).(x - y), (x + y).(x + y)] (*This finds the number of differences*)
                                                     (*between two state vectors x and y*)

(*recognizing the x and -x are the same*)

Correct[x_, y_] := 0 /; Error[x, y] == 0;
(*the vectors x and y are the same if error=0*)
Correct[x_, y_] := 1 /; Error[x, y] != 0;
SS1 = Si;                  (*Store the designated attractor of the sparce matrix as SS1*)
```

Choose Random Second Steady State (orthogonal)

```
differ = Size / 2;
(*Choose second designated attractor SS2 orthogonal to SS1*)
change = RandomSample[Range[Size], differ];  (*As SS1[i]=+/-1,
For SS1.SS2=0, SS1[i]=-SS2[i] at exactly 1/2 the postions*)
SS2 = SS1;
(*This section randomly chooses these positions and set the values of SS2*)
n = 1;
While[n ≤ differ,
 {indx = change[[n]],
  n = n + 1,
  SS2[[indx]] = -SS2[[indx]]}]
```

Construct New Matrix

```
t1 = Outer[Times, SS1, SS1];
(*Construct the connectivity mattrix for a system with 2 designated attractors*)
t2 = Outer[Times, SS2, SS2];        (*as per Hoppfield and store as matrix Int*)
int = (1 / 2) t1 + (1 / 2) t2;        (*int[i,j]=1/2 SS1[i]*SS1[j] + SS2[i]*SS2[j] *)

FixedConnections = {};
(*Initiailize List of connections inherited from skeleon connectivity matrix T*)
                                  (* that are not to be deleted*)

PossibleChoices = {};
(*Initiailize list of links added to T so that both SS1 and SS2 are now attractors*)
                                  (*These links may later be pruned*)


For[row = 1, row ≤ Size, row++,              (*For (i,j) such that T[ij]≠0: Uij=T*ij *)
 For[column = 1, column ≤ Size, column++,
  (*For (i,j) such that T*ij=0:Uij=(si*sj+ri*rj)/2 = Int[i,j]*)
  Choice = ((row - 1) * Size + column);
  If[int[[row, column]] != 0, PossibleChoices = Union[PossibleChoices, {Choice}]];
  If[oldmatrix[[row, column]] ≠ 0, {int[[row, column]] = t[[row, column]],
    FixedConnections = Union[FixedConnections, {Choice}]}]
 ]
]
```

```
Umatrix = int;
PossibleChoices = Complement[PossibleChoices, FixedConnections];
NumChoices = Length[PossibleChoices];
NumConections = Length[Union[PossibleChoices, FixedConnections]];
MatrixPlot[Umatrix]
```

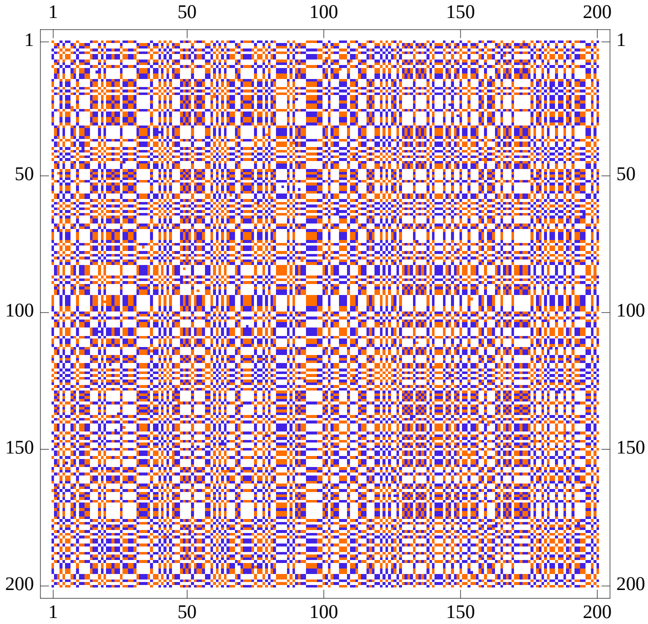

Next Remove Fixed Number of Links

```
testgood = 1;     (* a boolean variable will be set to 0 if the  *)
                  (* pruned network passes the test *)

avgtwostatespeed = 0;
fraction = 0;
testnum = 200;     (*number of random initial conditions to test*)
ittlimit = 300;    (*number of itterations to allow for convergence*)
NumToKeep = Round[Size * 15];
(* number of connections to keep =an average of 15 links per node *)
                                        (*connections ~ deleting the rest%*)
NumFixed = Length[FixedConnections];
While[testgood ≠ 0,
 {
  avgtwostatespeed = 0,
  tempU = IdentityMatrix[Size] - IdentityMatrix[Size],
  (*Create an array of 0's of proper size*)
  TempChoices = RandomSample[PossibleChoices, NumToKeep - NumFixed],
  (*Randomly choose conncections to be preserved*)
  AllTempConnections = Union[TempChoices, FixedConnections],
  (*Recognizing that this must include the skeleton T*)

  For[i = 0, i ≤ NumToKeep - 1, i++
    {Connection = AllTempConnections[[i]],
     (*For just these positons set TempU[i,j]=U[i,j]*)
     r1 = Quotient[Connection - 1, Size] + 1,   (*For the rest TemU[i,j]=0*)
     r2 = Mod[Connection - 1, Size] + 1,
     tempU[[r1, r2]] = Umatrix[[r1, r2]]}
  ],
  test = 1,
  etot = 0,
  totS1 = 0,     (*Will tally number of random tests that converge to SS1*)
  totS2 = 0,     (*Will tally number of random tests that converge to SS2*)
  While[(test ≤ testnum) && (etot == 0),
   {test = test + 1,
    tempvector[0] = Table[RandomReal[{-1, 1}], {i, 1, Size}],
    (*create random initial vector*)
    d = 1,
    (*d will be the distance between two subsequent *)
    n = 0,
    (*applications of the net, d=0 implies convergence *)
    While[(n < ittlimit) && (d > 0),
     (*loop until the max number of iterations completed*)

     (*or until the sequence has converged *)
         {n = n + 1,
          tempvector[n] = Hop[tempU, tempvector[n - 1]],
```

```
            d = Error[tempvector[n], tempvector[n - 1]]
            }],
      totS1 = totS1 + Abs[Correct[tempvector[n - 1], SS1] - 1],
      (*Update the convergence tallies*)
      totS2 = totS2 + Abs[Correct[tempvector[n - 1], SS2] - 1],
      c = Min[Correct[tempvector[n - 1], SS1], Correct[tempvector[n - 1], SS2]],
      (*will be 0 only if cnverged to SS1 or SS2*)
      etot = etot + c,
      avgtwostatespeed = avgtwostatespeed + n - 1,
      (*avgtwostatespeed is the number of iterations +1, required for*)

      (*convergence. It is a running total and needs to be divided *)
                                                  (*by the number of tests,
      to get the average num of iterations*)

      fraction = totS2 / (totS1 + totS2)
      (*fraction of initial conditions that converged to SS2*)
     }],

    If[etot == 0,                       (*If there were no errors, accept these deletions*)
     {Umatrix = tempU,                  (*and set boolean flag accordingly*)
      PossibleChoices = TempChoices,    (*else testgood=1 and will try again*)
      NumConections = Length[AllTempConnections],
      (*PossibleChoices now becomes the list of nodes eligible*)
      testgood = 0},                              (*For future deletion*)
     testgood = 1],
    Print[etot, " ", NumConections]
   }]
  Umatrix = SparseArray[Umatrix];
  twostatespeed = {};
  basin = {};
  basin2 = {};
  twostatespeed = Join[twostatespeed, {{NumConections, avgtwostatespeed / testnum}}]
  basin = Join[basin, {{NumConections, fraction}}]
  (*Record the size of network, and fraction of conditions*)
                                                 (*Converging to 2nd state*)
  basin2 = Join[basin2, {{NumConections / Size * 1.0, fraction}}]
```

Remove Links at Random checking to see if link already tested discounting links that are a node's only input

```
  Cnt = 0;
  TempChoices = PossibleChoices;
  (*A list of connections that might be removable in the present network configuration*)
  NumConections = Length[Union[PossibleChoices, FixedConnections]];
```

```
(*Total size of network*)
NumChoices = Length[PossibleChoices];
NumTempChoices = NumChoices;
removals = 0;
done = 0;
ittlimit = 300;
(*number of itterations to allow for convergence*)
testnum = 200;                                (*number of random initial conditions to test*)
Print["removals", " ", "NumTempChoices", " ", "NumChoices" ];


While[NumTempChoices > 0,                    (*Continue looping
  until there are no more removable links in the present configuration*)
 {
  removals = removals + 1,
  Choice = RandomChoice[TempChoices],        (*Choose random link from list for removal*)
  r1 = Quotient[Choice - 1, Size] + 1,
  (*and map back to row and column of matrix*)
  r2 = Mod[Choice - 1, Size] + 1,
  originalmatrixlement = Umatrix[[r1, r2]],   (*Store value of matrix elexment*)
  Umatrix[[r1, r2]] = 0,
  (*Temporarliy set elemet to 0, cut connection*)
  etot = 0,

  test = 0,                                   (*Number of initial conditions tested*)
  totS1 = 0,
  (*Running tally of conditions that converged to SS1 or SS2*)
  totS2 = 0,
  avgtwostatespeed = 0.,
  If[Hop[Umatrix, SS1] ≠ SS1, etot = 1],
  (*If the SS1 ans SS2 are not both fixed points of the map after*)
  If[Hop[Umatrix, SS2] ≠ SS2, etot = 1],       (*removing the connection,
  the connection cannot be severed*)

  While[(test ≤ testnum) && (etot == 0),
   (*loop until the required # tests have been run, or a test was failed*)
   {test = test + 1,
    tempvector[0] = Table[RandomReal[{-1, 1}], {i, 1, Size}], (*Random initial vector*)

    d = 1,
    (*d will be the distance between two consecutive itterations of the hopfield net *)
    n = 0,  (*d=0 implies the output has converged to a steady state*)

    While[(n < ittlimit) && (d > 0),  (*Continue to itterate
      map until it has converged or reached max number of itterations*)
        {n = n + 1,
         tempvector[n] = Hop[Umatrix, tempvector[n - 1]],
         d = Error[tempvector[n], tempvector[n - 1]]
         }],
    avgtwostatespeed = avgtwostatespeed + n - 1,
```

```
      totS1 = totS1 + Abs[Correct[tempvector[n - 1], SS1] - 1],
      (*Update # of initail vectors convergint to SS1 ans SS2*)
      totS2 = totS2 + Abs[Correct[tempvector[n - 1], SS2] - 1],
      c = Min[Correct[tempvector[n - 1], SS1], Correct[tempvector[n - 1], SS2]],
      (*c is 0 if the network converged to SS1 or SS2*)

      etot = etot + c,
      fraction = totS2 / (totS1 + totS2),
      If[d > 0, etot = 100]
      (*if the network hadnt converged to a steady state..the connection was essential*)
     }],

   If[(etot == 0),
    (*if the network did converge to SS1 or SS2 for all initial conditions*)
    {Umatrix[[r1, r2]] = 0,    (*delete link permanently and
      update lists of choices for future removal*)
     PossibleChoices = Complement[PossibleChoices, {Choice}],
     TempChoices = PossibleChoices,
     NumConections = NumConections - 1,
     NumChoices = NumChoices - 1,
     NumTempChoices = NumChoices,
     twostatespeed = Join[twostatespeed, {{NumConections, avgtwostatespeed / testnum}}],
     basin = Join[basin, {{NumConections, fraction}}],
     basin2 = Join[basin2, {{NumConections / Size, fraction}}]
    },
    {                                                  (*if the
      network did NOTconverge to SS1 or SS2 for all initial conditions*)
     Umatrix[[r1, r2]] = originalmatrixlement,     (*restore link to original value*)
     TempChoices = Complement[TempChoices, {Choice}],
     (*Remove this link from the list of links that might be removed in the*)
     NumTempChoices = NumTempChoices - 1              (*present configuration*)
    }
    (*note that if a different link is removed 1st so that network config changes*)
   ],
   (*this link will again be considered for removal*)

   If[Mod[removals, 60] == 0,                          (*update user as to progress*)
    {
     Print[removals, " ", NumTempChoices, " ", NumChoices ]
    }
   ]
  }]
Basinfile1 = "Basin" <> Sz <> ".mx"
Basinfile2 = "BasinNodes" <> Sz <> ".mx"
DumpSave[Basinfile1, basin];
DumpSave[Basinfile2, basin2];
```
